# Genome-Wide SNP Discovery and Mapping QTLs for Seed Iron and Zinc Concentrations in Chickpea (*Cicer arietinum* L.)

**DOI:** 10.3389/fnut.2020.559120

**Published:** 2020-10-15

**Authors:** Syed Sab, Ramappa Lokesha, D. M. Mannur, Somasekhar, Kisan Jadhav, Bingi Pujari Mallikarjuna, Laxuman C, Sharanbasappa Yeri, Vinod Valluri, Prasad Bajaj, Annapurna Chitikineni, AnilKumar Vemula, Abhishek Rathore, Rajeev Kumar Varshney, I. Shankergoud, Mahendar Thudi

**Affiliations:** ^1^Department of Genetics and Plant Breeding, University of Agricultural Sciences - Raichur (UAS-R), Raichur, India; ^2^Center of Excellence in Genomics and Systems Biology, International Crops Research Institute for the Semi-Arid Tropics (ICRISAT), Hyderabad, India; ^3^Zonal Agricultural Research Station, University of Agricultural Sciences - Raichur, Kalaburagi, India

**Keywords:** chickpea, seed, iron, zinc, SNP, QTL

## Abstract

Biofortification through plant breeding is a cost-effective and sustainable approach towards addressing micronutrient malnutrition prevailing across the globe. Screening cultivars for micronutrient content and identification of quantitative trait loci (QTLs)/genes and markers help in the development of biofortified varieties in chickpea (*Cicer arietinum* L.). With the aim of identifying the genomic regions controlling seed Fe and Zn concentrations, the F_2:3_ population derived from a cross between MNK-1 and Annigeri 1 was genotyped using genotyping by sequencing approach and evaluated for Fe and Zn concentration. An intraspecific genetic linkage map comprising 839 single nucleotide polymorphisms (SNPs) spanning a total distance of 1,088.04 cM with an average marker density of 1.30 cM was constructed. By integrating the linkage map data with the phenotypic data of the F_2:3_ population, a total of 11 QTLs were detected for seed Fe concentration on CaLG03, CaLG04, and CaLG05, with phenotypic variation explained ranging from 7.2% (*CaqFe3.4*) to 13.4% (*CaqFe4.2*). For seed Zn concentration, eight QTLs were identified on CaLG04, CaLG05, and CaLG08. The QTLs individually explained phenotypic variations ranging between 5.7% (*CaqZn8.1*) and 13.7% (*CaqZn4.3*). Three QTLs for seed Fe and Zn concentrations (*CaqFe4.4, CaqFe4.5*, and *CaqZn4.1*) were colocated in the “*QTL-hotspot*” region on CaLG04 that harbors several drought tolerance-related QTLs. We identified genes in the QTL regions that encode iron–sulfur metabolism and zinc-dependent alcohol dehydrogenase activity on CaLG03, iron ion binding oxidoreductase on CaLG04, and zinc-induced facilitator-like protein and ZIP zinc/iron transport family protein on CaLG05. These genomic regions and the associated markers can be used in marker-assisted selection to increase seed Fe and Zn concentrations in agronomically superior chickpea varieties.

## Introduction

Micronutrients are vital for healthy growth and development of the human body, and their adequate intake through diet is a prerequisite for various metabolic functions. However, a majority of the world's population face difficulties in meeting the recommended daily allowances (RDA). This has led to micronutrient malnutrition (also known as “hidden hunger”) that affects one in three people worldwide (1). Iron (Fe) and zinc (Zn) are important micronutrients that play a major role in the normal growth and development of humans by acting as cofactors for several proteins including hemoglobin, cytochrome, and transcription factors (2). Fe and Zn deficiency causes anemia, tissue hypoxia, dwarfism, lowered disease immunity, stunting, poor cognitive development, and orificial and acral dermatitis (3, 4). It is estimated that by 2050, around 1.4 billion women and children would be Fe deficient and 175 million zinc deficient (5). This risk will be high in Southeast Asia, Africa, and the Middle East. Micronutrient malnutrition has received increased global attention in recent decades, with efforts to address it by various strategies such as mineral supplementation and dietary diversification. Biofortification of staple crops through plant breeding is a highly cost-effective and long-term strategy to enhance micronutrient density in crop plants (6). Efforts in this direction have involved understanding the genetic architecture of Fe and Zn concentrations in seeds of cereals crops (7–10). More recently, the focus has been on pulses that serve as secondary staples, mainly because of their increased protein content (11). Among legumes, common bean (*Phaseolus vulgaris*), lentil (*Lens culinaris*), Pea (*Pisum sativum*), and mung bean (*Vigna radiata*) have been targeted for micronutrient studies (6).

Chickpea, a highly nutritious diploid (2*n* = 2*x* = 16) legume crop plant having a genome size of 738 Mbp (12) ranks second after dry beans in terms of global production among pulses, with over 14.77 million tons produced in 2017 (FAOSTAT, accessed on 17 February 2020). Chickpea is considered an excellent whole food and a source of carbohydrate (52–71%), protein (18–24%), fiber (10–23%), fat (4–10%), minerals and vitamins, dietary fiber, folate, β-carotene, and some important fatty acids (13, 14). Genomic research during the last decade enabled the decoding of the genome sequence (12) and sequencing of several germplasm lines (15–17) and provided insights into the genetics of complex abiotic and biotic stresses (18). Besides simplifying these complex traits, molecular breeding lines with enhanced drought tolerance and disease resistance were developed and released for commercial cultivation (19–21). Nevertheless, very few genomic studies have attempted to understand the variability for nutritionally important traits. The variability study (22) for physicochemical, nutritional and cooking quality traits among 30 chickpea germplasm lines indicated that higher 100-seed weight reduced cooking time. Using principal component analysis, variation for seed and flour characteristics among 79 chickpea germplasm accessions used in European breeding programs revealed higher protein and fiber in desi and higher fat content in kabuli types (23). Recently, studies on genotype and environment effects on seed quality traits demonstrated that environment plays an important role in variation in amylose and amylopectin content (24). None of these studies utilized genomics tools to understand the genetics of these traits.

Marker trait associations for protein content have been reported using simple sequence repeat markers (25). In the case of chickpea, there are very few studies on Fe and Zn accumulation in seeds. Genetic variations in the concentration of Fe (36.2–86.4 mg kg^−1^) and Zn (18.6–62.2 mg kg^−1^) were reported in 94 diverse accessions of chickpea (26). Fe and Zn in seeds appear to be a complex trait governed by a number of major genes/quantitative trait loci (QTLs), and the concentration of these minerals is highly influenced by edaphic and environmental factors (2). Recently, besides the variation in seed mineral concentration among cultivars and breeding lines (27, 28), markers linked with seed Fe and Zn content were reported in a select set of germplasm collections (2, 26). The identification of new sources of Fe and Zn concentrations, their genetic dissection, and using them to diversify the breeding population is a continuous process in developing high-yielding chickpea varieties with enhanced nutrition content. Cost-effective and high-throughput discovery and genotyping of single nucleotide polymorphism (SNP) markers using genotyping-by-sequencing (GBS) approach (29) have been widely used in many crop species including chickpea to assess diversity, in genome-wide association studies, in constructing high-density genetic linkage maps, and QTL identification (2, 30–33).

This study reports on high-throughput genome-wide marker discovery and genotyping using GBS approach and QTLs for Fe and Zn concentrations in chickpea, using an F_2:3_ intraspecific mapping population developed from MNK-1 × Annigeri 1.

## Materials and Methods

### Mapping Population and DNA Extraction

A mapping population consisting of 188 F_2_ plants was developed from a cross between MNK-1 and Annigeri 1. MNK-1 is a kabuli type, extra-large-seeded chickpea variety having Fe and Zn concentrations of 72.88 and 30.98 mg kg^−1^, respectively. Annigeri 1 is a desi-type, medium seed size variety having Fe and Zn concentrations of 98.67 and 37.07 mg kg^−1^, respectively. Fresh, young leaves were collected from parental lines, and each F_2_ individual and high-quality genomic DNA was extracted using high throughput NucleoSpin® 96 Plant II Kit (Macherey-Nagel, Düren, Germany) following the manufacturer's protocol. The DNA was quantified using a spectrophotometer (Shimadzu UV160A, Japan) and used to prepare the GBS library for SNP discovery. Individual F_2_ plants were selfed, and seeds harvested were advanced to develop an F_2:3_ population for phenotyping seed Fe and Zn contents.

### Genotyping-by-Sequencing and SNP Calling

Genotyping by sequencing was carried out for SNP calling between the parental genotypes and genotyping the 188 F_2_ progenies following the protocol described by Elshire et al. (29). GBS libraries were prepared by restriction digestion of DNA of each of the F_2_s as well as the parents with *ApeKI* endonuclease (recognition site: G/CWCG), followed by ligation with uniquely barcoded adapters using T4 DNA ligase. An equal proportion of barcoded adapters ligated DNA fragments from each sample were mixed to construct the GBS libraries, which were amplified and purified in order to remove excess adapters, followed by sequencing on the HiSeq 2500 platform (Illumina Inc., San Diego, CA, USA). The sequence reads from fastq files were used for SNP identification using GBSv2 (34) pipeline implemented in TASSEL v5 (35). In short, the sequencing reads were first searched for barcode information and reads with no Ns. The barcode containing reads was then demultiplexed according to the barcode sequence and trimmed. The remaining good quality and distinct reads (tags) were retrieved in the form of fastq and aligned against the draft genome sequence of chickpea (36) using Bowtie2 (37). The alignment file was then parsed through the remaining GBSv2 pipeline for SNP calling and genotyping. The SNPs identified were then filtered in order to retain data with contrasting alleles in parental genotypes and those having <30% missing data for further analysis.

### Phenotyping for Seed Fe and Zn

During the crop season of 2019, F_2:3_ progenies (plant:progeny rows) were planted following augmented design at the Zonal Agricultural Research Station, Kalaburagi, Karnataka (latitude, 17.36 and longitude, 76.82). Each experimental block consisted of a single 2-m row plot with 30 cm spacing between rows and 10 cm interplant spacing. The experimental plot was divided into six blocks to reduce heterogeneity, and each occupied 27 lines plus 4 checks (MNK-1, Annigeri 1, KAK-2, and BGD-103) replicated twice in each block. Standard cultivation practices were followed to ensure healthy crop growth.

Mature seeds of each parental line and mapping individual (five to six representative plants from each F_2:3_ progeny line) were collected separately during harvesting stage. Care was taken to avoid the contamination of seeds with dust and inert particles during harvest and while sample preparation. Five grams of dry seed sample (with 5.6% moisture content) from each accession was ground into fine powder, and 0.5 g of fine ground powder was put in a reaction vessel to which was added 10 ml of di-acid mixture (nitric acid and hydrochloric acid in a 9:1 ratio). The vessel was sealed and loaded into the chamber and microwave of the Microwave Digestion System (Anton Paar Company, USA), which was set to run for 40 min at 180°C. After the vessels cooled down to <50°C, they were removed from the chamber and moved to an exhaust clean hood to vent excess pressure slowly. The digested samples from the vessel were then placed in a clean container and diluted with distilled water to make up a volume of 50 ml in a conical flask. They were then analyzed for Fe and Zn concentrations. An atomic absorption spectrophotometer (Analytik Jena AG, contrAA 700, Germany) was used to estimate Fe and Zn concentrations based on the absorption of light at a wavelength of 370 and 388 nm, respectively. Both Fe and Zn concentrations were estimated and expressed as mg kg^−1^ (ppm) seed.

### Statistical Analysis

Variance analysis was done using PROC MIXED of SAS v9.4 (38), considering blocks and checks as fixed and F_2:3_ progeny lines as random. Repeated checks were used to estimate error mean square. Best linear unbiased predictors (BLUPs) and best linear unbiased estimates (BLUEs) were estimated for random and fixed factors, respectively. Genetic variability parameters including mean, range, frequency distribution, phenotypic coefficient of variance (PCV), genotypic coefficient of variance (GCV), broad sense heritability [h^2^_(bs)_], and genetic advance per mean (GAM) were computed using SAS.

### Genetic Linkage Map Construction

The allele calls for all genotypes were compiled and used for genetic linkage analysis by employing JoinMap V4.1 program (39). In total, 185 F_2_s were used to construct the genetic map after discarding three individuals with insufficient data. Chi square goodness-of-fit test was performed (*p* < 0.05) to test the segregation distortion for each marker. Highly distorted and unlinked markers were excluded from the analysis. Regression mapping algorithm with maximum recombination frequency of 0.3 at minimum logarithm of odds (LODs) of 3 and jump threshold of 5 were used to group the markers into eight linkage groups (LGs). Ripple command was used after adding each marker locus to confirm marker order. Map distance was estimated using Kosambi's mapping function (40). The final high-resolution linkage map was generated using LinkageMapView package in R software (41).

### QTL Analysis

The genotyping data of the intraspecific linkage map and phenotypic data for iron and zinc content from the F_2:3_ population were used for QTL analysis using QTL Cartographer V2.5 software (42). Composite interval mapping (CIM) method was used by selecting Model 6 with the default window size of 10 cM, control marker number 5, and using backward stepwise regression. For each trait, the LOD score threshold was determined by performing 1,000 permutations at significance level of *p* ≤ 0.05. The amount of phenotypic variation explained was determined using the coefficient of determination (*R*^2^) value and expressed as phenotypic variation explained (PVE) percentage.

### Identification of Genes in QTL Regions

The QTL region's coordinates were compared to the reference genome's gff file using bedtools (43) to identify the genes lying in the QTL region and functionally categorized using UniProt KB database (http://www.uniprot.org/).

## Results

### Genetic Variability for Seed Fe and Zn Concentrations

**Figure 1:**
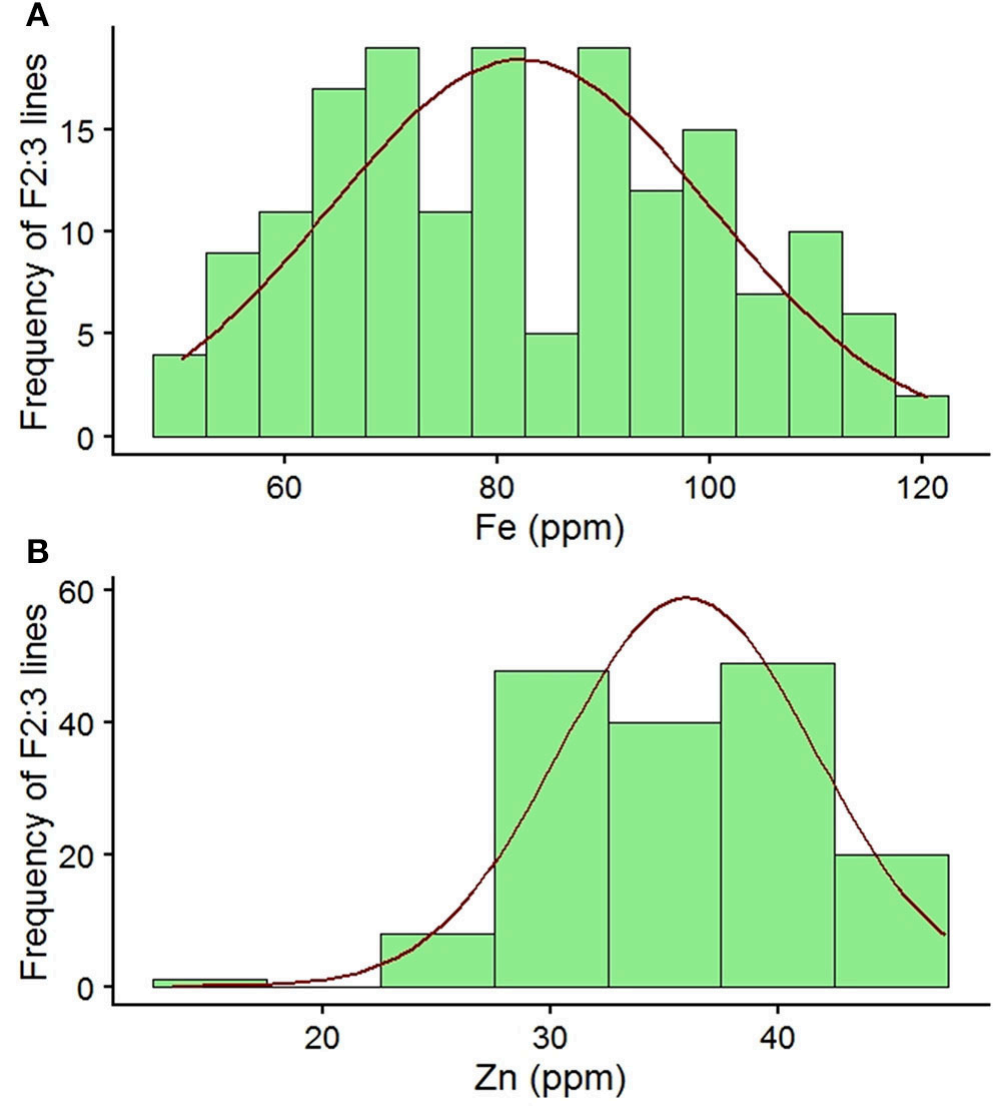
**(A, B)** Frequency distribution of seed Fe and Zn concentrations among F_2:3_ individuals of an intraspecific mapping population (MNK-1 × Annigeri 1) along with checks and parental lines.

The parental lines of the mapping population had wide variation for seed Fe and Zn concentrations. Parent MNK-1 had seed Fe and Zn concentrations of 72.88 and 30.98 mg kg^−1^, respectively, while parental line Annigeri 1 had seed Fe and Zn concentrations of 98.67 and 37.07 mg kg^−1^, respectively. The F_2:3_ population showed wide variability for both the traits (Table 1, Figure 1). Seed Fe concentration ranged from 51.60 to 118.74 mg kg^−1^ with a mean of 82.15 mg kg^−1^. Seed Zn concentration ranged from 17.89 to 45.60 mg kg^−1^ with a mean of 36.10 mg kg^−1^. Analysis of variance (ANOVA) results indicated highly significant genotypic effects (Table 2). A high GCV (21.89%) and PCV (22.17%) for seed Fe concentration and moderate GCV (14.07%) and PCV (15.11%) for seed Zn concentration were observed among the F_2:3_ progenies. Very high broad sense heritability values of 97.50 and 86.68% were observed for seed Fe and Zn concentrations, respectively.

**Table 1:** Phenotypic variation for seed iron and zinc concentrations in F_2:3_ generation of the cross MNK-1 × Annigeri 1.

**Traits**	**Mean**	**Range**		**GCV (%)**	**PCV (%)**	**h^2^_**(bs)**_ (%)**	**GAM (%)**
		**Min**.	**Max**.				
Seed Fe	82.15	51.60	118.74	21.89	22.17	97.50	44.45
Seed Zn	36.10	17.89	45.60	14.07	15.11	86.68	26.92

*GCV, genotypic coefficient of variance; PCV, phenotypic coefficient of variance; h^2^_(bs)_, broad sense heritability; GAM, genetic advance per mean*.

**Table 2:** Variance component for F_2:3_ progeny lines and F-statistic for block, checks, and check vs. progeny lines for seed Fe and Zn concentrations ANOVA for augmented design in F_2:3_ generation of the cross MNK-1 × Annigeri 1.

**Source of variation**		**Seed Fe**	**Seed Zn**
**Random effect**	**Df**	**VC**	**VC**
F_2:3_ progeny lines	161	323.52**	25.78**
**Fixed Effect**	**Df**	**F-statistic**	**F-statistic**
Block	5	0.37	1.69
F_2:3_ progeny lines vs. Checks	1	0.002	0.53
Checks	3	92.59**	24.25**
Error	15	8.30	3.96

*VC, variance component for random factors*.

** *Significant at prob < 0.01*.

### Identification of SNPs Using GBS Approach and Construction of Intraspecific Linkage Map

**Figure 2:**
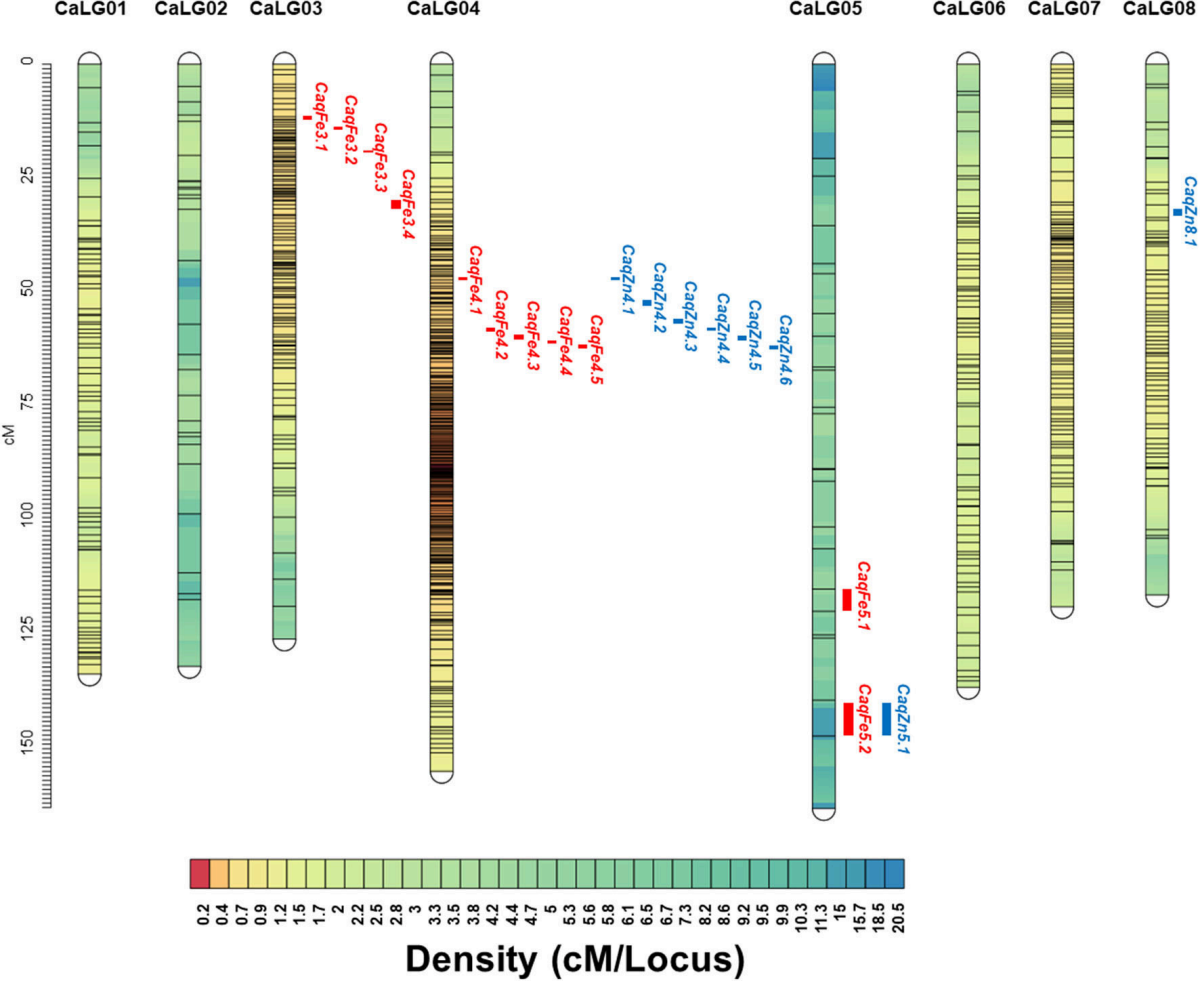
Intraspecific genetic linkage map of chickpea constructed using the F_2_ population derived from the cross MNK-1 × Annigeri 1. The linkage map with eight linkage groups (CaLG01–CaLG08) shows the quantitative trait loci (QTLs) for seed Fe and Zn concentrations; 11 QTLs for seed Fe and 8 QTLs for seed Zn concentration are shown on the left side of the corresponding linkage group.

Genotype by sequencing of the parental genotypes generated ~211.46 Mb for MNK-1 and ~144.44 Mb for Annigeri1. For 188F_2_s, ~46.6 Gb was generated with average of ~257.76 Mb per sample. A total of 31,311 SNPs were identified, of which 7,204 (23%) were polymorphic between MNK-1 and Annigeri 1, and the remaining 17,034 (54.40%) were monomorphic. A set of 7,204 polymorphic SNPs was used for linkage map analysis. As a result, a total of 839 SNPs were mapped on the eight chickpea chromosomes postfiltration of SNPs' set (Supplementary Table 1). A total of 7,204 SNPs from GBS data were used to generate the intraspecific linkage map. Of these, 839 SNPs were grouped into eight linkage groups (CaLG), whereas the remaining 6,365 SNPs were unlinked to any of the linkage groups. Marker density per linkage group varied from 24 (CaLG05) to 318 (CaLG04), with an overall average of 104.8 markers per linkage group (Table 3, Figure 2). The final linkage map had a total length of 1,088.04 cM, with CaLG05 (164.17 cM) being the largest and CaLG08 (116.98 cM) being the smallest. The average intermarker distance in the linkage map was 1.30 cM, with CaLG04 being the densest (0.49 cM) and CaLG05 being the least dense (6.84 cM). The final linkage map is shown in Figure 2.

**Table 3:** Features of an intraspecific genetic map based on the F_2_ population of the cross MNK-1 × Annigeri 1.

**Linkage group**	**Polymorphic markers**	**Markers mapped**	**Map length (cM)**	**Intermarker distance (cM)**
CaLG01	1,126	76	134.56	1.77
CaLG02	625	28	132.68	4.74
CaLG03	866	138	126.92	0.92
CaLG04	1,081	318	155.80	0.49
CaLG05	917	24	164.17	6.84
CaLG06	1,184	75	137.32	1.83
CaLG07	1,179	108	119.60	1.11
CaLG08	226	72	116.98	1.62
Total	**7,204**	**839**	**1,088.04**	**1.30**

### QTL Mapping for Seed Fe and Zn Concentrations

Genotyping data of mapped 839 SNPs was integrated with the phenotypic data of the Fe and Zn concentrations and analyzed using composite interval mapping (CIM) method. For seed Fe concentration, a total of 11 QTLs were mapped on CaLG03, CaLG04, and CaLG05 with LOD thresholds ranging from 3.0 (*CaqFe3.4* and *CaqFe5.2*) to 4.8 (*CaqFe4.2*) (Table 4, Figure 2). PVE by individual QTLs ranged from 7.2% (*CaqFe3.4*) to 13.4% (*CaqFe4.2*). At all the five loci on CaLG04 (*CaqFe4.1, CaqFe4.2, CaqFe4.3, CaqFe4.4*, and *CaqFe4.5*), alleles from parent MNK-1 favored seed Fe concentration. For seed Zn concentration, eight QTLs were detected on CaLG04, CaLG05, and CaLG08 (Table 4, Figure 2). QTLs *CaqZn5.1* and *CaqZn8.1* had the lowest LOD threshold of 3.0, whereas QTL *CaqZn4.3* had the highest LOD score of 6.3. The PVE of these QTLs ranged between 5.7% (*CaqZn8.1*) and 13.7% (*CaqZn4.3*). At all the loci except *CaqZn5.1*, the alleles from parent MNK-1 favored seed Zn concentration.

**Table 4:** Quantitative trait loci (QTLs) for seed Fe and Zn concentrations in F_2:3_ population.

**Trait**	**QTL**	**Linkage group**	**Left marker**	**Right marker**	**Position (cM)**	**LOD**	**Additve variance**	**Phenotypic variation explained (PVE; %)**
Seed Fe	*CaqFe3.1*	CaLG03	SCA3_38112971	SCA3_31331006	12.81	4.2	21.2	9.3
	*CaqFe3.2*	CaLG03	SCA3_38103368	SCA3_38060814	15.51	4.1	22.4	8.0
	*CaqFe3.3*	CaLG03	SCA3_36227565	SCA3_36549464	20.41	4.5	19.8	9.7
	*CaqFe3.4*	CaLG03	SCA3_39612119	SCA3_37949465	32.91	3.0	16.1	7.2
	*CaqFe4.1*	CaLG04	SCA4_11398699	SCA4_11244334	48.91	3.1	−12.9	9.8
	*CaqFe4.2*	CaLG04	SCA4_23943928	SCA4_13840227	59.21	4.8	−17.0	13.4
	*CaqFe4.3*	CaLG04	SCA4_13787448	SCA4_13845581	61.11	4.0	16.6	11.6
	*CaqFe4.4*	CaLG04	SCA4_13724666	SCA4_4477846	62.61	3.3	−14.3	11.6
	*CaqFe4.5*	CaLG04	SCA4_13840191	SCA4_3223962	63.81	3.4	−15.5	11.2
	*CaqFe5.1*	CaLG05	SCA5_40736291	SCA5_47154065	119.81	4.2	4.7	7.4
	*CaqFe5.2*	CaLG05	SCA5_37174786	SCA5_35312501	145.31	3.0	15.2	10.5
Seed Zn	*CaqZn4.1*	CaLG04	SCA4_11398699	SCA4_11244334	48.91	4.4	−7.0	11.4
	*CaqZn4.2*	CaLG04	SCA4_12558541	SCA4_11441673	53.41	4.6	−6.8	6.5
	*CaqZn4.3*	CaLG04	SCA4_13660846	SCA4_13787649	57.61	6.3	−8.1	13.7
	*CaqZn4.4*	CaLG04	SCA4_23943928	SCA4_13840227	59.21	6.0	−7.9	13.0
	*CaqZn4.5*	CaLG04	SCA4_13787448	SCA4_13845581	61.11	5.5	−8.0	12.1
	*CaqZn4.6*	CaLG04	SCA4_13840191	SCA4_3223962	63.81	4.6	−7.7	10.7
	*CaqZn5.1*	CaLG05	SCA5_37174786	SCA5_35312501	146.31	3.8	5.2	10.2
	*CaqZn8.1*	CaLG08	SCA8_4106644	SCA8_16027922	33.81	3.8	−6.8	5.7

### Colocalization of Seed Fe and Zn Concentration QTLs and Genes in the QTL Regions

Based on the physical position of the SNP markers linked with the seed Fe and Zn QTLs, we found that three QTLs (*CaqFe4.4, CaqFe4.5*, and *CaqZn4.1*) are colocalized in the “*QTL-hotspot*” region that harbors several drought tolerance related QTLs on CaLG04, as reported earlier (Table 5). We identified a total of 7,496 genes in the QTL regions reported in the study (Supplementary Table 2). Among these, we identified three genes—Ca_20872, Ca_00798, and Ca_01283—in the *CaqFe3.1* QTL region that encode for iron–sulfur assembly protein IscA-like 1, iron–sulfur cluster assembly protein IscA, and Zn-dependent alcohol dehydrogenase family class III protein, respectively. Further, gene Ca_04513 that encodes for iron ion binding oxidoreductase was identified in the *CaqFe4.4* QTL region. Interestingly, we identified gene Ca_01633 that encodes for ZIP zinc/iron transport family protein in the QTL region of *CaqZn5.1*.

**Table 5:** Colocalization of Fe and Zn Quantitative trait loci (QTLs) in the “*QTL-hotspot”* region reported by Varshney et al. (44).

**Fe/Zn QTL reported in this study**				**“*****QTL-hotspot*****” region**				
**Pseudomolecule**	**Start**	**End**	**QTL**	**Pseudomolecule**	**Start**	**End**	**QTL**	**Overlap**
Ca4	4477846	13724666	*CaqFe 4.4*	Ca4	13239546	13378761	“*QTL-hotspot_a”*	139215
Ca4	3223962	13840191	*CaqFe 4.5*	Ca4	13239546	13378761	“*QTL-hotspot_a”*	139215
Ca4	3223962	13840191	*CaqZn4.1*	Ca4	13239546	13378761	“*QTL-hotspot_a”*	139215
Ca4	4477846	13724666	*CaqFe4.4*	Ca4	13393647	13547009	“*QTL-hotspot_b”*	153362
Ca4	3223962	13840191	*CaqFe4.5*	Ca4	13393647	13547009	“*QTL-hotspot_b”*	153362
Ca4	3223962	13840191	*CaqZn4.1*	Ca4	13393647	13547009	“*QTL-hotspot_b”*	153362

## Discussion

Growing hunger and high levels of different forms of malnutrition are major challenges to achieving food and nutritional security. Effective interventions like biofortification aimed at guaranteeing access to nutritious foods are needed to address micronutrient malnutrition or hidden hunger. In several pulse crops, biofortification through plant breeding has gained momentum in the past decade (6). In chickpea, knowledge of genetic variation underlying the concentration of important seed minerals such as Fe and Zn and their association with yield-related traits is vital for accelerating breeding for biofortified varieties. In the present study, an F_2:3_ population of chickpea was used to identify QTLs for seed Fe and Zn concentrations.

The parental lines MNK-1 and Annigeri 1 had seed Fe concentrations of 72.88 and 98.67 mg kg^−1^ and seed Zn concentrations of 30.98 and 37.07 mg kg^−1^, respectively. The seed Fe concentration values of the cultivars are comparatively higher than the range of 49–56 mg kg^−1^ in chickpea cultivars reported by Vandemark et al. (28). With respect to seed Zn concentration, the parental values are comparable (35–43 mg kg^−1^) to Vandemark et al. (28) but higher than the range of 21–28 mg kg^−1^ as reported by Ray et al. (27) for chickpea cultivars. Jayalaxmi et al. (45) reported that Fe concentration varied from 26 to 146 mg kg^−1^ and Zn concentration from 35 to 77 mg kg^−1^ in 56 chickpea varieties. Analysis of variance **(**ANOVA) results inferred a highly significant difference among F_2:3_ individuals for both seed Fe and Zn concentrations (Table 2). The seed Fe and Zn concentrations were in the range of 51.60–118.74 and 17.89–45.60 mg kg^−1^, respectively (Table 1). It is evident from the distribution graph that F_2:3_ individuals recorded values beyond the checks for both seed Fe and Zn concentrations (Figure 1). In addition, a very high broad sense heritability was observed among the F_2:3_ individuals for both seed Fe [h^2^_(bs)_ = 97.50] and Zn [h^2^_(bs)_ = 86.68] concentrations. This indicates the feasibility of early generation selection that may result in improving these traits. Given that there are reports of significant environment effects on the concentration of minerals (26, 27) and this study was conducted only at a single location, better genotype × environment can be realized by further evaluating the advanced generation population across different locations or years.

Identification of genetic variants like SNPs is a key step in several genetic studies, including linkage mapping and QTL identification. Among the different genetic variants, SNP markers have become the markers of choice due to their high abundance and cost effectiveness. GBS provides increased efficiency in SNP discovery and genotyping by enabling high multiplexing of samples and simple library preparation procedures (29). This method involves only a targeted sequencing approach and does not require the complete genome sequencing of all individuals of the mapping population and parental genotypes. Due to its high-throughput efficiency, GBS has been used in recent studies in SNP identification and QTL mapping for important traits in chickpea (33, 46, 47). This study employed the GBS approach to identify SNPs in an intraspecific F_2_ mapping population derived from two popular cultivars of chickpea. As a result, 7,204 novel SNPs were identified and used for genetic linkage analysis. The SNPs identified in this study were higher compared to other GBS studies in chickpea (33, 46) but lower than those reported by Deokar et al. (47). Of the 7,204 SNPs used, 839 were mapped on to eight linkage groups covering a total map distance of 1,088.04 cM and average marker density of 1.30 cM. The map density is comparable with that of Jaganathan et al. (33) and less dense compared to that of Hiremath et al. (48) and Deokar et al. (47, 49). Of these 839 SNPs, a majority (37%) were mapped on CaLG04. Earlier, Jaganathan et al. (33) made similar observations that may be attributed to the presence of high repeat-rich regions in Ca4 pseudomolecule of chickpea as reported by Varshney et al. (36).

The intraspecific linkage map was utilized to identify QTLs linked to seed Fe and Zn concentrations. A total of 11 QTLs for seed Fe concentration were detected on CaLG03, CaLG04, and CaLG05, which explained phenotypic variance in the range of 7.2–13.4%. For seed Zn concentration, eight QTLs were identified on CaLG03, CaLG05, and CaLG08. The phenotypic variance explained by these QTLs ranged between 5.7 and 13.7%. Previous studies have reported QTLs for seed Fe on chromosomes 1, 4, and 6 (26) and on chromosomes 1, 3, and 4 (2) and for seed Zn on chromosomes 1, 4, and 7 (26) and on chromosomes 2 and 3 (2). In all these studies, the distribution of QTLs on multiple chromosomes clearly indicates the quantitative nature and complexity of seed Fe and Zn concentration traits.

In this study, several colocated QTLs for seed Fe and Zn concentrations were identified on CaLG04 and CaLG05. For instance, QTLs *CaqFe*4.1-*CaqZn*4.1, *CaqFe*4.2-*CaqZn*4.2, *CaqFe*4.3-*CaqZn*4.1, and *CaqFe*4.5-*CaqZn*4.1 were colocalized on CaLG04 in the same marker interval SCA4_11398699-SCA4_11244334, SCA4_23943928-SCA4_13840227, SCA4_13787448-SCA4_13845581, and SCA4_13840191-SCA4_3223962, respectively (Table 4, Figure 2). On CaLG05, QTLs *CaqFe*5.1-*CaqZn*5.1 were colocalized within the SNP marker interval of SCA5_37174786-SCA5_35312501. Previous studies have reported QTL colocalization for micronutrient concentration in chickpea on chromosomes 4, 5, and 7 (2) and also in rice (10). Hence, these genomic regions on chromosome 4 may contain conserved QTLs for seed Fe and Zn concentrations across diverse genetic backgrounds. Pleiotropy or tight linkage of genes controlling multiple traits may be the reason for the colocalization of QTLs (50). These regions with colocated QTLs will be useful for simultaneous improvement using marker-assisted selection (10).

Interestingly, QTLs for seed Fe and Zn were also mapping in the “*QTL-hotspot region*” reported earlier (44). This genomic region was successfully introgressed into different elite genetic backgrounds, and molecular breeding lines with enhanced climate resilience have been reported (36). The molecular breeding lines may also be rich in seed Fe and Zn, as the QTLs are in the genomic region that was introgressed. By comparing the QTL region's coordinates to the kabuli reference genome of chickpea (12), the identified genes were found to be involved in Fe- and Zn-related activities. The F_2:3_ population exhibited wide variation for seed Fe and Zn concentrations. A high heritability was obtained for both the traits studied.

This study was successful in using the GBS approach to develop a linkage map for an intraspecific population in chickpea. The map consisted of 839 SNPs spanning 1,088.04 cM. Genomic regions for seed Fe and Zn concentrations were mapped on multiple linkage groups, and the linked SNPs were reported. These QTLs are good candidates for further validation, functional characterization, and gene cloning to clarify the role of these genes in Fe and Zn homeostasis in chickpea. The SNPs linked to seed Fe and Zn concentrations have potential for use as informative molecular tags in marker-assisted breeding to improve seed Fe and Zn concentrations in chickpea varieties.

## Data Availability Statement

All datasets generated for this study are included in the article/Supplementary Material.

## Author Contributions

IS and MT conceived the idea. SS conducted the experiments, analyzed the data, and prepared the manuscript along with BM. AV and AR performed statistical analysis. VV and PB did the GBS analysis. RL, DM, LC, SY, and KJ contributed to the germplasm characterization and mapping population development and phenotyping. AC and RKV contributed consumables and to the generation of genotyping data. All authors contributed to the article and approved the submitted version.

## Conflict of Interest

The authors declare that the research was conducted in the absence of any commercial or financial relationships that could be construed as a potential conflict of interest.
